# Practice

**Published:** 1889-08

**Authors:** S. W. Cohen

**Affiliations:** Waco, Texas


### PRACTICE.

S. W. Cohen, M. D., Waco, Texas.

'Tis difficult to think of the two terms, practice and therapeutics, independently of one another, as they carry the same idea throughout.

Whilst we may differentiate by very closely shading the interpretation of these two words, very little license is necessary to use them interchangeably. Practice may possibly comprehend a larger scope of utilities, as applicable to disease, but accepting either term as we may, the medical world has always associated them with the employment of certain substances or certain methods, or both, in aborting, modifying, or curing abnormal mental or physical conditions, recognized by such special names as diarrhœa, neuralgia, pneumonia, melancholia, mania, amentia, etc. A combination of seemingly similar symptoms was arbitrarily designated by certain appellations. The *name* of the disease was prescribed for by old-school physicians, with well-known formulæ, indorsed by some acknowledged authority, or in late years, and in our own school, with certain drugs, singly or in alternation, without any special care having been taken to individualize each particular case and prescribe for the sum total of symptoms instead of for certain denominated conditions which had been so labeled in the interests of automatic prescription.

To many practitioners of our special mode of practice—and 'tis pleasant to recognize a goodly number of such present—practice, therapeutics, materia medica, surgery, obstetrics—excluding, of course, necessary manual or mechanical interference in the latter two branches named—in fact, all of medicine, as taught under a true, conscientious, and experienced disciple of Samuel Hahnemann, is so intimately associated that it is difficult to *separate* it into branches.

Practice, therefore, to a simon-pure homœopathist, but signifies the attack of the totality of symptoms (no matter what conditions or circumstances may influence) by the simillimum, without regard to an arbitrary diagnosis. Practice to an advanced physician of the homœopathic school means simply the utilization of a well-searched materia medica at the bedside, under the guidance of the law of similars. Ætiology, pathology, diagnosis, and prognosis, certainly have a scientific worth, *as such*, but the most erudite medical lecturer, who is wont to "paralyze" a class of gentlemen with six-syllabled, triple-jointed, hard medical names, and whose delicately convoluted brain is a store-house of knowledge in *these* fields, is oftentimes an indifferent prescriber And why? Because symptomological and not pathological or physiological prescription is the foundation-stone of Homœopathy. A physician may sit by the bedside, and impress his trusting, but, as far as medical matters are concerned, agnostic patient, with the extraordinary fund of his knowledge as to his (the patient's) condition. He may *dilate* by the hour, and yet his patient may *die soon*, because the doctor's knowledge only extended far enough to diagnose the disease, while he had not the ability to discover the simillimum. 'Tis a difficult task, 'tis true, to ignore our frequent spontaneous diagnosis, and too often does this diagnosis suggest the remedy —*i. e.*, the drug or drugs for this general condition to which has been affixed a diagnostic conclusion, while the individualization of each distinct case is lost sight of. Homœopathic is a misnomer for such practice.

It is not good practice (because it is not homœopathic practice) to prescribe Aconite whenever we recognize a fever, nor Aconite and Bryonia in alternation when we diagnose a pneumonia, nor Belladonna and Mercurius for every sore throat. 'Tis to be hoped we all indorse this declaration, but do we not know that just such practice is very common (outside of Texas, of course), and, therefore, so many failures have been recorded against Homœopathy.

A very recent case in my own practice may be utilized to illustrate the influence brought to bear upon one's prescription, and if in this case, I stand self-condemned, I will endeavor to bear all reproaches with humility. I was called to see Mr. R. A. M., æt. thirty-four. Rheumatism! Yes, there is that element of disturbance, the diagnosis. Still all discernible objective symptoms were closely observed, and the subjective ones elicited to the best of my ability. The pains were located chiefly in the right arm, between the shoulder and elbow, and in the right hip-joint, thigh, and knee-joint. Severe frontal headache. These and other symptoms were aggravated by motion, and were worse at night. Bryonia. Called next day, March 23d. General condition unchanged. Pains still very severe, but had shifted to left knee-joint and hip, and the left arm and shoulder were also slightly affected. Closer questioning elicited the following: "I was taken with pains while on the cars," said the patient. "I sat in my seat until I could sit no longer. On arising my pains became *very* severe, and continued for a few moments, while I was moving about, but soon the constant motion brought relief; further continued motion now in its turn brought back the pains, and I was forced to seek relief by rest. It was a constant alternation between motion and quietude to obtain even transient alleviation. Warm applications are grateful." Rhus tox. certainly seemed indicated, and it was the remedy I prescribed. Patient was somewhat easier on the third day. Continued the remedy at longer intervals. No apparent change on the fourth day. Sac. lac. On the fifth day the pains had shifted back to his right side. The pains in his arm were not so severe, but he was suffering very acutely with the nether limb. He was "flighty." Would start suddenly from short naps; would see visions, and every jar, even a step upon the floor, would aggravate his sufferings. Belladonna was prescribed. By the morning of the 28th inst., the sixth day of his illness, the last noted symptoms had disappeared, but the pains in the right limb were excruciating.

The patient *must* have the limb (which was now resting on a pillow) moved, but to even touch it, let alone to disturb its position, was torture to the poor fellow, whose cries and groans were heartrending, and whose flood of tears brought a suspicious moisture into my own eyes. I was in a quandary, so I left some Sac. lac. and went home to study up the case. Returned to the patient that evening with a *Materia Medica*, a *Repertory* and voluminous notes. The patient was, if anything, suffering more than ever, and had not slept for at least forty hours. He begged for "just a little Morphine." His wife, who is as staunch an allopathic adherent as he is a homœopathic one remarked, "Doctor, *my* physician would not permit *me* to suffer so." Something had to be done. My patient was certainly in a worse condition than I found him on my first visit, and I feared the old-school prescription of six weeks in bed would prove the only efficacious one. I sat close up to the bedside, fortified with my notes and reference books to take the case afresh. Impressed with the shifting character of the pains, Pulsatilla had attracted my attention, but still I placed my confidence in Rhus. I now began to inquire on the line of Pulsatilla, careful, though, to ask no leading questions, with the following result: Pains continually shifting back and forth, though worse in right hip, thigh, and knee. The patient had not taken two drinks of water during the six days of his confinement. Had been somewhat nauseated every day, and had vomited once. He is the mildest mannered gentleman in our glorious city of Waco, and of complexion the fairest, his eyes being light-blue and his moustache and hair almost flaxen. Without referring to my volumes or notes, I arose and placed one dose of Pulsatilla$^{cm}$ on his tongue, promising him speedy relief. Sac. lac. in water, was to be given every half-hour, while he was awake. As I stepped into his chamber next morning he was all smiles. Within three hours after I left him he was entirely free from pain, and could turn over, something he was unable to do up to this time. He had slept soundly all night, and was very comfortable indeed, only a little stiff and sore. Some fresh Sac. lac. was prepared, to be taken once every two hours. There was no return of the pains, the patient was discharged next day, and he was on the street the following Monday, two days after. Here I must record decided failures with Bryonia, Rhus tox., and Belladonna. Rhus seemed to somewhat mitigate the symptoms, and the symptoms Belladonna was prescribed for disappeared in a few hours, but still neither proved the simillimum. It may be possible that we may ameliorate the most pressing and painful symptoms by presenting a drug that covers the most urgent indications, and zig-zag our way through unknown labyrinths of drug action until we perhaps relieve the patient, but the prominent cause of such miserable work depicted above is due to the fact that our practice demands that not only must we prescribe for the totality of symptoms, but that the eleven more *prominent*, *uncommon*, and *peculiar* (characteristic) features of the case (see § 153, *Organon*) must be especially considered. Had a Hering, a Lippe, or a Dunham prescribed for Mr. M., his week of torture might have been very appreciably curtailed. Another case in which the prescription was more fortunate is worth detailing.

Was called to see J. B., æt. ten. Had been ill six days, and under the care of an old-school doctor. The history and symptoms present at my first call pointed to another diagnosis, pneumonia.

Temperature 104°. Circumscribed pain in lower portion of right thorax; face flushed; short respiration, and very painful. Constant cough, which was still more painful, and the little patient endeavors to suppress it. Viscid sputa. Breathing entirely thoracic. Dull sound on percussion. I employed every method to obtain the exact condition present, but did not permit myself to be guided by pathological reasonings. Bryonia was presented. On my next visit, the following day, the temperature was found to be 104½° an exacerbation of one-half degree, and no improvement in any respect was observable. The little fellow was picking at his fingers and lips continually. His pillow was bespecked with blood that had been rubbed from his lips during the night. Lips sore and raw. Tongue felt sore, and there was a large ulcer-like abrasion on the edge of his tongue on the right side, a little more than half-way back. His throat felt raw, and everything he put into his mouth "burned" him. His nose appeared stuffy. He received but one single dose of Arum. triph.[c], dry upon the tongue (sent him from my office at eleven A. M.), and Sac. lac. every hour. At my next morning's visit I received the following account from the mother:

"My little boy was free of fever by three o'clock yesterday evening, and has had none since. He slept well all night, and has ceased picking at his fingers and lips. He has but little cough."

I inquired from the patient regarding the tongue and throat symptoms and they had entirely vanished, the abrasion on the tongue being almost healed. The child did not cough during my visit. I discharged the case, and he has remained well to date. With the symptoms detailed the little fellow would have recovered just as rapidly if Arum had been given, no matter what the pathological condition or diagnosis. As homœopaths we must conform our practice to the spirit and letter of the master's words, as given us in the *Organon*, for in the pages of this inspiration we find the method—the *true* and *only* method—of the application of medicinal substances to diseased conditions.

The foregoing was written some time in April, in anticipation of our State meeting, which was to have taken place May 7th and 8th, but was postponed to June 4th and 5th. On reading the "Proceedings of the Lippe Society of Philadelphia" in my May, 1889, number of THE HOMŒOPATHIC PHYSICIAN, I found on page 179 the following Pulsatilla symptom as given by Dr. James: "*The slightest motion aggravated, yet he was forced to move the leg. Must get a new position yet there was no relief.*" This was a prominent symptom in the first case quoted, but not until I saw the May number of THE HOMŒOPATHIC PHYSICIAN

was I aware of the fact that my prescription so completely covered it. [This symptom of Puls. was given us by the venerable Dr. Lippe in one of his numerous instructive conversations with us during the many years we enjoyed his friendship.—W. M. J.]

